# Hemorrhagic cystitis in allogeneic stem cell transplantation: a role for age and prostatic hyperplasia

**DOI:** 10.1007/s00520-022-06916-8

**Published:** 2022-02-18

**Authors:** Eugenio Galli, Federica Sorà, Luca Di Gianfrancesco, Sabrina Giammarco, Elisabetta Metafuni, Maria Assunta Limongiello, Idanna Innocenti, Francesco Autore, Luca Laurenti, Patrizia Chiusolo, Andrea Bacigalupo, Simona Sica

**Affiliations:** 1 Sezione di Ematologia, Dipartimento di Scienze Radiologiche Ed Ematologiche, Università Cattolica del Sacro Cuore, Rome, Italy; 2 Dipartimento di Diagnostica per Immagini, Radioterapia Oncologica ed Ematologia, Fondazione Policlinico Universitario A. Gemelli IRCCS, Rome, Italy; 3 Dipartimento Di Scienze Mediche E Chirurgiche, Fondazione Policlinico Universitario A. Gemelli IRCCS, Rome, Italy

**Keywords:** Allogeneic stem cell transplantation, Hemorrhagic cystitis, Prostatic hyperplasia

## Abstract

**Purpose:**

Hemorrhagic cystitis (HC) is a frequent complication of allogeneic hematopoietic stem-cell transplantation (HSCT). HC worsens transplant outcomes and patient wellbeing in terms of pain, hospitalization, and need for supportive care. A deeper understanding of the risk factors of HC may lead to more intensive prevention in high-risk patients.

**Methods:**

In this report, we analyzed 237 consecutive patients who received HSCT with the aim of identifying possible risk factors for HC and their consequences, with a particular focus on transplant- and gender-related risk factors.

**Results:**

HC occurred in 17% of patients, with a higher incidence in males (21% vs 11%, *p* = 0.03). Risk factors identified for HC included age over 55 years, male recipient, HLA mismatch, reduced intensity conditioning, and cyclophosphamide-based graft-versus-host disease (GVHD) prophylaxis. Increased HC was seen in patients with grade II–IV acute GVHD and detectable BKV and JCV viruria. In a multivariate model, increased age remained significant (*p* = 0.013). Patients with HC had longer hospitalizations and increased non-relapse mortality (NRM). Among male recipients, independent risk factors for HC included age (*p* = 0.016) and prostate volume (*p* = 0.016). Prostatic hyperplasia (volume more than 40 cm^3^) occurred in 33% of male patients, of which 32% developed HC (compared with 16% of patients without prostatic hyperplasia; *p* = 0.032).

**Conclusions:**

Age is the most important risk factor for HC. Additional potential risk factors include cyclophosphamide-based GVHD prophylaxis and HLA mismatch. Among male recipients, prostatic hyperplasia is an additional independent risk factor. As HC is common and associated with prolonged hospitalization, more intensive prophylactic strategies should be considered in high-risk patients.

## Introduction

Allogeneic hematopoietic stem cell transplantation (HSCT) is a curative cell therapy for patients with hematologic malignancies. Conditioning chemotherapy may be myeloablative (MAC) or reduced-intensity (RIC), according to diagnosis, stem cell source, and the general condition of the patient. Different regimens are known to affect the duration and severity of cytopenias, and in some cases, the durability of disease control [1]. After infusion of stem cells, acute or chronic graft-versus-host disease (a/c-GVHD) may be observed with variable grades of severity. Causes of non-relapse mortality (NRM) include multiorgan toxicity, infections, and hemorrhages occurring during pancytopenia, as well as GVHD. While usually not fatal, hemorrhagic cystitis (HC) is another commonly observed toxicity of HSCT. As the risk factors and optimal management of HC are not well defined, HC may be considered an unmet need that heavily impacts the post-transplant hospitalization.

The incidence of HC varies between 12.2% and 36.9%, and the median occurrence is within the first 30 days. In the literature, the major risk factors for HC reported include age (in pediatrics), myeloablative conditioning regimen, and haploidentical or mismatched HLA donor [2–4]. A comparison of incidence of HC in matched vs. haploidentical or mismatched transplants in 122 patients uniformly treated with post-transplant cyclophosphamide (PTCY)-based GVHD prophylaxis confirmed that HLA matching is a risk factor independent of immunosuppressive regimen (Copelan 2019). In a multivariate analysis of 161 haploidentical transplants, risk factors for HC included myelosuppressive conditioning and use of tacrolimus vs. cyclosporine [5]. Gender has also been investigated as a possible risk factor for HC, though there is no consensus among studies.

Cyclophosphamide has toxic effects on the bladder mucosa, and both cyclophosphamide-containing conditioning regimens and cyclophosphamide-based GvHD prophylactic regimens have been associated with increased HC [6, 7]. Consequently, the predominant use of cyclophosphamide-based GvHD prophylaxis in haploidentical transplants compared with matched transplants may represent an important source of bias.

While there are some studies reporting a higher incidence of HC in males with benign prostatic hypertrophy [8], benign prostatic hypertrophy as a possible risk factor for HC in the setting of HSCT has not been investigated.

In the present study, we aimed to investigate the difference in incidence, severity, and duration of HC between males and females, and we aimed to evaluate the role of prostatic hypertrophy in the development of HC among other commonly tested risk factors. Our hypothesis was that prostatic hypertrophy might have put some male patients at higher risk of HC through augmented urinary retention and possible chemical/infectious damage.

## Patients and methods

We retrospectively collected data on all 237 consecutive patients who underwent HSCT in our Institution from December 2017 to December 2020. Hematopoietic Cell Transplantation-Comorbidity Index (HCT-CI) was calculated for all patients at the moment of hospitalization for HSCT [9]. We collected data on patients’ characteristics as primary disease, transplant conditioning regimen, donors age, and HLA matching (Table 1).

**Table 1:** Characteristics of patients of both genders with uni- and multi-variate analysis for HC. Major hematological and demographic characteristics of patients are shown in this table and compared to HC-FS through Cox analysis. Only variables with univariate p values of 0.05 or less acceded to multivariate analysis

		Patients	Without HC n(%)	With HC n(%)	Univariate analysis p value	Multivariate analysis p value	HR (95%ci)
Total		237	197 (83)	40 (17)			
Diagnosis	AML	101	84 (83)	17 (17)			
	ALL	30	27 (90)	3 (10)			
	MPN	54	41 (75)	13 (25)			
	NHL	17	15 (88)	2 (12)			
	HL	3	3 (100)	0 (0)			
	AA	5	3 (60)	2 (40)			
	MDS	27	24 (89)	3 (11)			
Chronic vs Acute diseases	HL/NHL/MPN/AA	79	62 (78)	17 (22)	0.19		
	AML/ALL/MDS	158	135 (85)	23 (15)			
HCT-CI	Missing data	7			0.33		
	0–1	67	58 (87)	9 (13)			
	> 1	163	133 (82)	30 (18)			
Recipient age	Median	56	55	61.5	**0.002**		
	Up to 55	109	102 (94)	7 (6)	** < 0.001**	**0.013**	**3.06 (1.26–7.39)**
	Over 55	128	95 (74)	33 (26)			
Recipient gender	Females	102	91 (89)	11 (11)	**0.034**	0.25	1.56 (0.73–3.33)
	Males	135	106 (79)	29 (21)			
HLA matching	MSD	53	50 (94)	3 (6)			
	MUD	102	87 (85)	15 (15)			
	Haplo	75	54 (72)	21 (28)			
	CB	7	6 (86)	1 (14)			
Matched vs haplo	MSD and MUD	155	137 (88)	18 (12)	** < 0.001**	0.11	1.94 (0.86–4.35)
	Haplo	75	54 (72)	21 (28)			
Conditioning regimen	Missing	6					
	TT-Bu-Flu	190	156 (82)	34 (18)			
	Baltimore	24	19 (79)	5 (21)			
	Flu-TBI	17	17 (100)	0 (0)			
	Other	5	3 (60)	2 (40)			
RIC vs MAC	MAC	180	154 (86)	26 (14)	**0.032**	0.54	1.2 (0.62–2.44)
	RIC	51	38 (75)	13 (25)			
GVHD prophylaxis	Triple MTX based	43	42 (98)	1 (2)	**0.02**	0.068	6.6 (0.86–47.5)
	Triple PTCY based	188	150 (80)	38 (20)			

Legend: *AML*, acute myeloid leukemia; *ALL*, acute lymphoid leukemia; *MPD*, myeloproliferative neoplasm; *NHL*, non Hodgkin lymphoma; *HL*, Hodgkin lymphoma; *AA*, aplastic anemia; *MDS*, myelodysplastic syndrome; *HCT-CI*, Hematopoietic cell transplantation-specific comorbidity index; *MSD*, matched sibling donor; *MUD*, matched unrelated donor; *TT-Bu-Flu*, Thiotepa, busulfan and fludarabine; *Flu-TBI*, Fludarabine and Total Body Irradiation; *MAC*, myeloablative conditioning regimen; *RIC*, reduced intensity conditioning regimen; *GVHD*, graft versus host disease; *MTX*, methotrexate; *PTCY*, post transplant cyclophosphamide. Statistical significance is highlighted in bold

Prophylaxis for GVHD consisted of a triple regimen including cyclosporine and mycophenolate mofetil and either post-transplant cyclophosphamide (PTCY) or methotrexate. Apart from patients with severe aplastic anemia, from April 2019 onward, a uniform PTCY-based GvHD prophylactic regimen was used for all patients, due to center choice and expertise. Acute and chronic GVHD (aGVHD and cGVHD) were evaluated according to current criteria on a scale of 0 to 4 and 0 to 3, respectively [10].

Hemorrhagic cystitis was considered as any manifestation of macroscopic hematuria, from pinkish urine to massive clots, or acute anemia requiring urgent cauterization or intervention, corresponding to grade 2 to 4 hematuria according to current literature criteria [11, 12]. BKV and JCV were assessed by performing PCR before conditioning and following onset of urinary symptoms or HC, with the highest obtained values used in statistical analysis.

Prostate volume and diameters were assessed in males by an expert urologist with pre-HSCT imaging reviewed when available. Manual segmentations and delineations of the prostate was outlined without seminal vesicles. The prostate volumes were measured by calculating prolate ellipse volume by means of the (height × length × width × π/6) formula. In order to minimize interobserver-dependent variations and selection and measurement biases, all the measurement were performed by the same urologist who was blinded to whether the patients did experience HC or not; the prostate volume quantification was performed by the same imaging technique, namely computed tomography (CT), as trans-rectal ultrasound (TRUS) was not available for all patients, and it could be expected to be more operator–dependent than CT. However, in a recent systematic review, prostate imaging with TRUS and CT was found to be sufficiently accurate when quantitative measurements of prostate gland volume are required [13]. Prostatic hyperplasia was radiologically considered grade 0 when up to 20 cm^3^, grade 1 if 20–40 cm^3^, grade 2 if 40–60 cm^3^, grade 3 if 60–80 cm^3^, and grade 4 if more than 80 cm^3^ in volume [14].

Hemorrhagic cystitis-free survival (HC-FS) was considered as time from transplantation to first episode of HC, death, or last follow-up.

The effect of patient-related risk factors, including age, gender, HCT-CI and prostate volume in male patients, and procedure-specific risk factors, including HLA matching, GVHD prophylaxis, and conditioning regimen, on incidence and timing of HC was assessed in univariate and multivariate Cox regression analysis. The effect of parametric and non-parametric categorical variables and continuous variables on HC-FS was assessed using univariate and multivariate Cox regression analysis. Differences in median values were evaluated using Welch’s test for unequal variances and Student’s t test. Survival was compared using Kaplan Meier curves. Non-relapse mortality (NRM) was assessed by calculating cumulative incidence functions. Statistical analysis was performed with NCSS Statistical Software.

## Results

Almost all patients underwent HSCT for hematologic malignancies. The stem cell source was predominantly matched unrelated and haploidentical donors. Patients and transplants characteristics are described in Table 1.

The majority of patients (76%) received a myeloablative conditioning regimen (MAC) consisting of thiotepa-busulfan-fludarabine (TT-Bu-Flu) [15] or fludarabine and 12 Gy total body irradiation (Flu-TBI); the remaining 24% of patients received a reduced-intensity conditioning (RIC) regimen consisting of fludarabine, cyclophosphamide, and 2 Gy TBI (Baltimore) or TT-Bu-Flu with reduced doses of busulfan.

The median onset of HC was day 8 after stem cell infusion. Patients who developed HC had a higher median age (61.5 vs. 55 years, *p* = 0.002). Using a cutoff of 55 years to define elderly patients (in accordance with the median age of our patient cohort), there was an increased risk of HC in elderly patients (26% vs. 6%, *p* < 0.001). An increased risk of HC was also seen in male patients vs. female patients (21% vs. 11%, *p* = 0.03). At day + 30, HC-FS was increased in females compared with males (91% vs. 80%, *p* = 0.03), with a plateau after the first month.

HC occurred in a similar proportion of patients with acute malignancies and patients with chronic diseases, including myeloproliferative neoplasms and lymphomas (15% vs. 22%; *p* = 0.19). There was also no difference in incidence of HC according to HCT-CI. Decreased HC-FS was seen in patients receiving RIC compared with MAC (14% vs. 25%, *p* = 0.03). Worthy of note, no patients treated with Flu-TBI (MAC regimen) experienced HC; in this subset, all patients were under 45 years old and had a diagnosis of ALL.

To investigate the effect of HLA matching on incidence of HC, we compared HC in HLA identical (sibling and unrelated) vs. haploidentical transplants. Incidence of HC was higher in haploidentical transplants (28% vs. 12%, *p* < 0.001). In detail, the incidence of HC was 6%, 15%, and 28% for sibling, matched unrelated, and haploidentical donors, respectively.

Patients who received GVHD triple prophylaxis with PTCY had an increased incidence of HC compared with patients who received triple GVHD prophylaxis with MTX (20% vs. 2%; *p* = 0.02). Of note, HC occurred in two out of five patients (40%) with severe aplasia.

In a multivariate model, age more than 55 years was an independent risk factor for HC (HR 3.06; 95% CI 1.26–7.39).

Overall, grade 0–1 or 2–4 acute GVHD occurred in 195 (82%) and 39 (18%) patients, respectively. The incidence of HC was decreased in patients with grade 0–1 aGVHD, compared with those with grade 2–4 aGVHD (13% vs 29%, *p* = 0.011).

To investigate the effect of BKV and JCV infections on HC, we assessed patients for BKV and JCV viruria. BKV and JCV viruria was positive at some point in hospitalization, respectively, in 18% and 39% of patients. Patients with no viral reactivation had a lower incidence of HC compared with those with detectable BKV or JCV in urine (15% vs. 33% *p* = 0.006 for BKV; 13% vs. 25% *p* = 0.022 for JCV). Among the 29 patients with urine positive for both BKV and JCV, 12 (41%) developed HC. We found no correlation between prostatic hyperplasia and detection of BKV or JCV in urine.

**Fig. 1:**
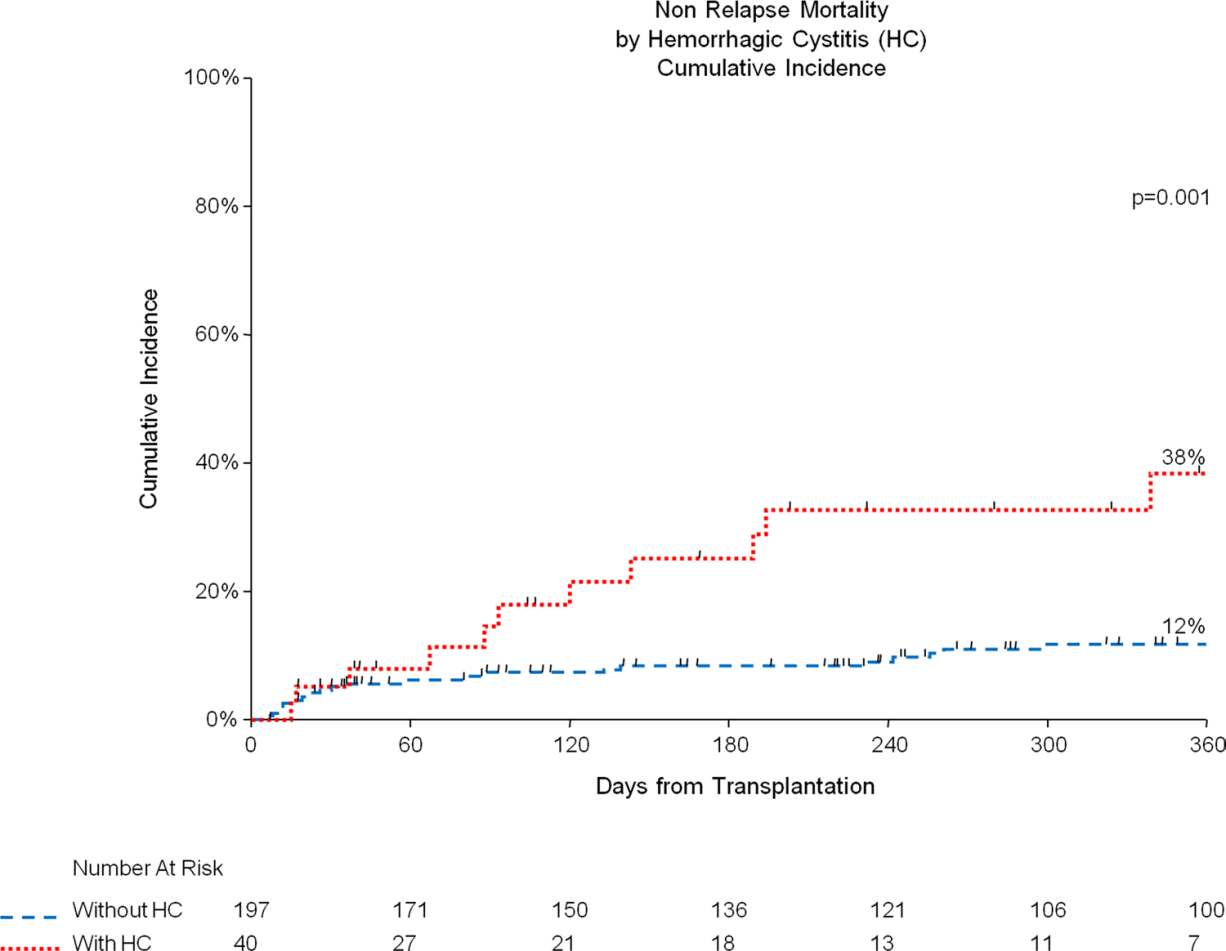
Non relapse mortality (NRM) and hemorrhagic cystitis (HC). Patients with HC (in red) had higher NRM compared to those without HC (in blue), with 8% vs 25% at 6 months and 12% vs 38% at 1 year (*p* = 0.001)

Median duration of HC was 7 days (range 1–85 days). Treatment of HC consisted of adequate hydration and platelet transfusions for all patients; some patients required more intensive treatment, which included continuous bladder irrigation (55% of patients with HC), specific antiviral therapy with cidofovir (7%), endoscopic diathermocoagulation (10%), and intravesical instillation of Platelet-Rich Plasma (3%) or hyaluronic acid (3%). Non-relapse mortality was significantly higher for patients with HC (*p* = 0.001), both 6 months (8% vs. 25%) and 1 year (12% vs. 38%) after transplant (Fig. 1). In patients who were successfully discharged, median length of hospital stay was increased in those who developed HC (36 vs. 26 days, *p* = 0.05).

Within male patients, we also focused on the potential effect of prostate volume on HC. Median prostate volume was evaluable in all but 16 male patients and it was greater in patients who developed HC (25 vs 32 cm^3^, *p* = 0.02). HC was increased in patients with prostatic hyperplasia of grade 2 and higher (volume more than 40 cm^3^), compared with patients with prostate volume less than 40 cm^3^ (32% vs. 16%; *p* = 0.03). As expected, age and prostate volume were strongly correlated (*p* < 0.001). Male patients with normal prostate volume (less than 40 cm^3^) had similar incidence of HC when compared to female population (*p* = 0.28, data not shown). In a multivariate model, increased HC was seen in patients with age greater than 55 years (*p* < 0.001). HLA mismatch (*p* = 0.008), and PTCY-based triple GVHD prophylaxis (*p* = 0.04); age greater than 55 years and prostate volume were independent risk factors for HC (*p* = 0.016).

## Discussion

In HSCT patients, hematuria is generally considered to be related to toxic or infectious insults to the urinary mucosae until proven otherwise, and “hematuria” and “hemorrhagic cystitis” are commonly used interchangeably. In this study, we aimed to evaluate how patient and transplant characteristics affect the development of HC.

In our cohort, HC was strongly associated with age, both increasing age as a continuous variable and age greater than 55 years compared with age less than 55 years. To the best of our knowledge, there are no other published studies investigating age as a risk factor for HC in an exclusively adult population. In a large retrospective study that included 1321 patients of all ages, age less than 20 years old was identified as a risk factor HC [12]; on the other hand, in a prospective study including 450 patients, no significant difference in HC was seen between patients less than 18 years (21%) and patients greater than 18 years (12.2%) [3].

Cyclophosphamide has been described as having a causative role in the development of HC when used in conditioning regimens [12]. The underlying cause of this association is acrolein, a cyclophosphamide urinary metabolite with toxic effects on the bladder mucosa. While some data are available on the risk of HC in patients treated with PTCY, a comparison between triple GvHD prophylactic regimens with post-transplant cyclophosphamide or methotrexate was not previously reported. In our patient cohort, we found that patients treated with PTCy had an increased incidence of HC, though this effect was not independent from age.

Moderate-to-severe aGVHD was associated with increased HC in our patient cohort: this is in partial disagreement with what was described in 2015 in a small French cohort, where 22 out of 33 haploidentical transplants treated with PTCY experienced HC independent of GVHD [7]. Our findings would be consistent with a role for GVHD in damaging the urothelium and, in conjunction, the direct or indirect effects of GVHD therapy consisting of high dose steroids and immunosuppression.

Matched transplants had a lower incidence of HC compared with haploidentical transplants. This is similar to the findings of Copelan et al. in 122 patients uniformly treated with PTCY-based prophylaxis, where they reported HC in 25% and 42% of patients, respectively, with HLA-matched and haploidentical transplants. In that study, the authors hypothesized that HLA-mismatched donor T cells could impair host antigen presenting cells, thus favoring BKV infections [4]. A strong association between HC and BKV was similarly found in our cohort of patients and was independent of other risk factors (data not shown). Oltolini et al. also reported that HLA mismatch favored early viral, but not bacterial, infections in 235 patients who received PTCY-based GVHD prophylaxis, [16]. While this immunological hypothesis is intriguing, it does not account for the effect of HLA mismatch on HC in patients who are BKV negative.

Data on the role of gender in development of HC is sparse and conflicting. Gargiulo et al. found no difference in incidence of HC in a prospective population of 450 mixed pediatric and adult patients of both genders (13% vs. 11%), while Lunde et al. found in a retrospective study of 1321 consecutive patients that male gender was associated with higher incidence of HC (15% vs 23%, *p* = 0.01) [3, 12]. In our study, male patients had a two-fold higher incidence of HC compared with females. We found that the risk of HC increased with increased prostate volume increased and that a cut-off of 40 cm^3^ was able to discriminate two populations with significantly different risk of HC (32% vs. 16%, *p* = 0.03). While there is a seemingly obvious association between age and prostatic hyperplasia, the two variables remained independently associated with HC in multivariate analysis. Moreover, male patients with prostate volume less than 40 cm^3^ had similar incidence of HC compared to female population, enforcing the hypothesis that the prostate may play a part in influencing the higher incidence and severity of HC in male patients. We hypothesize that toxic metabolites (e.g., acrolein) may have prolonged contact with bladder mucosa due to urinary retention in patients with prostatic hyperplasia. This exposure could reasonably contribute to chemical damage and bleeding in a thrombocytopenic and immunocompromised patient. Moreover, urinary retention may enhance viral damage from JCV and BKV on the urothelium. Prostate samples from patients with prostate cancer (PC) and prostatic hyperplasia have been found to be a reservoir of BKV and JCV in 22–32% of cases as well as a possible site of viral replication [17, 18]. As viral reactivation in the urinary tract is common in several immunodeficiency states, particularly for BKV, and polyomavirus in the urinary tract may contribute to HC, it is reasonable to hypothesize that prostatic hyperplasia, viral reactivation, and HC may be interconnected [19, 20].

Overall, patients with HC experienced worse clinical outcomes. Not only was HC associated with more prolonged hospitalization, but it was also associated with higher probability of NRM. HC was most frequently the cause of a prolonged hospital stay, as the acute management requires invasive procedures and intensive transfusion support. Post-transplant multi-organ toxicity is associated with infections, severe cytopenias, and renal failure; thus, HC is only the tip of the iceberg.

HC prophylaxis remains an area of debate. Some prophylactic approaches have been proposed based on prospective studies by the Gruppo Italiano Trapianto di Midollo Osseo (GITMO) and the European Conference on Infections in Leukemia (ECIL) guidelines on BKV-related HC, though the efficacy of these approaches has not been clearly demonstrated. In these reports, practices such as coadministration of Mesna with cyclophosphamide, urine alkalinization, intravenous hyper-hydration, and bladder continuous irrigation are addressed, while a role for antibiotic prophylaxis remains unclear [3, 21]. In 2008, Hadjibabaie et al. performed continuous bladder irrigation on 40 consecutive patients receiving sibling donor HSCT and cyclophosphamide-based conditioning and found that the incidence and duration of HC was decreased in these patients receiving prophylactic irrigation, compared with a historical cohort (32% vs. 50%, *p* = 0.1; 10 days vs. 18 days) [22]. In our population, three patients were treated with continuous bladder irrigation due to a history of intolerance to Mesna or previous HC, and none developed HC. While current management of HC is not formally standardized in the setting of allogeneic transplants, most patients are treated with intravenous hyper-hydration and intensive platelet transfusions (as time from transplant and platelet recovery seem to play a central role), and continuous bladder irrigation is used in only 12–27% of patients according to center experience [12, 21]. A limited amount of patients requires intravesical cauterization or intravesical instillation of hyaluronic acid, fibrin glue, or platelet-rich plasma. When viral BKV is detected, systemic or local specific therapy is indicated and can be curative in up to 70% cases. The possible roles of BKV genotype in and JCV in guiding HC prophylaxis are areas for further investigation [23, 24].

Data on prostatic hyperplasia have never been reported in the setting of HSCT with PTCY. A prospective evaluation of post-void residual urine and measurement of acrolein and viruria may clarify the pathogenesis of HC and thereby inform prophylactic management.

We recognize that our findings may present some limitations, especially concerning the retrospective nature of data collection, the missing data on prostate volume in some male patients and some variable transplant- and patient-related characteristics that may confound the analysis, as stem cell source and primary disease. We tried to overcome these limitations with multivariable risk analysis, while at the same time we remark the need for prospective trials in order to definitely explore risk factors and best prophylactic strategies for HC.

In conclusion, age over 55 years is an independent risk factor for hemorrhagic cystitis in patients treated with allogeneic stem cell transplantation. Mismatched transplants and PTCY-based triple GVHD prophylaxis may influence risk but are not independent of age. For male patients, prostatic hypertrophy may be an additional risk factor. More intensive prophylactic strategies may represent a reasonable option to prevent HC in the setting of transplantation in high-risk adults.

## Data Availability

For access to data, please contact the corresponding author.

## Code availability

Analysis was performed using NCSS Statistical Software.
